# The Impact of a Messenger-Based Psychosocial Chat Counseling Service on Further Help-Seeking Among Children and Young Adults: Longitudinal Study

**DOI:** 10.2196/43780

**Published:** 2023-05-17

**Authors:** Sabrina Baldofski, Elisabeth Kohls, Zeki Efe, Melanie Eckert, Shadi Saee, Julia Thomas, Richard Wundrack, Christine Rummel-Kluge

**Affiliations:** 1 Department of Psychiatry and Psychotherapy Medical Faculty Leipzig University Leipzig Germany; 2 Department of Psychiatry and Psychotherapy University Leipzig Medical Center Leipzig Germany; 3 krisenchat gGmbH Berlin Germany

**Keywords:** online intervention, e-mental health, online chat, hotline, text-based, children, adolescents, young adults, psychopathology, help-seeking

## Abstract

**Background:**

Mental crises have high prevalences in adolescence. Early interventions appear to be highly important to diminish the risk of the deterioration, recurrence, or chronification of symptoms. In recent years, various providers have started offering live chat support in psychological crises. The messenger-based psychological counseling service krisenchat aims to support young people in crises and, if necessary, provide a recommendation for a referral to the health care system or to seek further help from a trusted adult person.

**Objective:**

This study aimed to investigate the impact of using the counseling service of krisenchat on the further help-seeking behavior of young people, and to identify associated factors of further help-seeking.

**Methods:**

This longitudinal study analyzed anonymous data from 247 individuals who used krisenchat between October 2021 and March 2022, and received a recommendation for further help-seeking. An online survey directly after the chat assessed the perceived helpfulness of the chat and well-being after the chat. After 4 weeks, further help-seeking, facilitators and barriers to help-seeking, and self-efficacy were assessed in an online follow-up survey.

**Results:**

The most frequently recommended services or persons to seek further help from included a psychotherapist or social psychiatric service (75/225, 33.3%), a school psychologist or school social worker (52/225, 23.1%), and the user’s parents (45/225, 20.0%). Of the 247 users, 120 (48.6%) indicated that they contacted the recommended service or person, and of these, 87 (72.5%) stated that they already had an appointment (or talk) with the respective service or person or that an appointment (or talk) was scheduled. The most frequently reported facilitators for further help-seeking were mental health literacy (54/120, 45.0%), improvement of self-efficacy (55/120, 45.8%), and symptom recognition (40/120, 33.3%). In users not displaying further help-seeking behavior, the most frequent barriers included stigmatization (60/127, 47.2%), lack of mental health literacy (59/127, 46.5%), need for self-reliance and autonomy (53/127, 41.7%), and negative family beliefs regarding help services (53/127, 41.7%). Subgroup comparisons indicated significantly higher levels of self-efficacy in users displaying further help-seeking behavior than in those not displaying further help-seeking behavior. Both subgroups did not differ in gender, age, recommended service or person, chat topics, perceived helpfulness, and well-being.

**Conclusions:**

The findings of this study indicate that children and young adults receiving counseling on krisenchat benefit in terms of seeking further help. Further help-seeking seems to be associated with higher levels of self-efficacy.

**Trial Registration:**

Deutsches Register Klinischer Studien DRKS00026671; https://tinyurl.com/4fm5xe68

## Introduction

Several mental health conditions develop or have their onset at a young age, and their prevalence increases dramatically during adolescence [1,2]. The estimated worldwide prevalence of mental health problems of 10% to 20% among adolescents shows that this age group is affected more than any other age group [3,4]. Mental health problems thus represent an important health concern for young people [4,5]. Untreated symptoms are likely to persist and result in a compromised mental health status, for example, untreated affective disorders like depression are directly associated with suicidal behavior [6]. Besides the fact that the mortality rate of suicide increases with progressing teenage years, suicide is one of the leading causes of death among young people aged 10 to 24 years [6-8]. Therefore, early intervention to diminish the risk of the deterioration, recurrence, or chronification of symptoms appears to be especially important [9,10]. Nevertheless, help-seeking behavior and treatment use are low among the majority of adolescents and young adults with mental conditions. Thus, oftentimes, adolescents do not receive professional treatment [11-13].

The help-seeking process can be described as a stage model involving (1) an awareness of symptoms and an appraisal that support or treatment is needed, (2) the expression of symptoms and the need for help, (3) an awareness of available and accessible sources and services of help, and (4) the willingness to disclose concerns and problems to the selected source [14]. Besides the mere help-seeking process, it might be necessary to address young people’s willingness and readiness to reach out to a help service that is most suited to their needs [15]. This seems especially important as studies have shown that young people are overall more hesitant to seek help from mental health services [16,17]. Additionally, the more severe the symptoms, the less likely young people are to seek help [16,17]. For example, only about 1 in 5 German students with diagnosed anxiety or mood disorders had ever used mental health services in the past [18].

The low help-seeking behavior could potentially be explained by barriers to accessing mental health services. Systematic reviews have identified key barriers to help-seeking for mental health problems in young people, including the fear of stigma, family reactions or negative family beliefs toward mental health services, a lack of mental health literacy, the need for self-reliance and autonomy, concerns about confidentiality, and other barriers such as structural factors like access, time, transport, or cost [13,19].

Considering these barriers, mental health literacy might be a key factor in initiating help-seeking behavior. Mental health literacy refers to the ability to recognize, manage, and prevent mental health problems and includes the awareness of available sources of help [19,20]. Therefore, mental health literacy is an important step in adaptive coping and getting access to appropriate health care for mental health concerns [16,21]. Further, studies show that mental health services often provide information about mental health, leading to an increased mental health literacy [22]. In turn, people with an increased mental health literacy show a lower self-stigmatization regarding mental health problems, are more capable of recognizing their mental health status, and are more likely to seek help from appropriate and professional help services [23-26]. Moreover, the literature suggests that interventions focusing on mental health literacy significantly increase the readiness, motivation, and intention to seek help [27].

Young people prefer to solve their problems on their own, thus expressing a high need for self-reliance and autonomy [16,28]. The common use of digital media indicates that young people use the internet to find their own way to solve problems, and studies also show an increase in the self-reliant search for information or help to cope with mental health problems [14,29,30]. Regarding the above-mentioned barriers to help-seeking, internet-based services have many advantages, such as no geographical boundaries, usually free access, and anonymity and privacy [31], and could thus present an opportunity for low-threshold access to encourage further help-seeking behavior. Despite the increasing number of these services, there is a lack of systematic evaluations in the current literature.

One of the services offered is *krisenchat*, a German messenger-based psychological counseling service for children, adolescents, and young adults. The first cross-sectional evaluation showed a high acceptance, feasibility, and user satisfaction of this service [32]. The results indicated a high need for a 24/7 accessible, anonymous, and low-threshold online help service to offer rapid stress relief in acute crises such as suicidality [33]. Besides its goal to increase the mental health literacy of users, *krisenchat* aims to refer users in need to professional help services or personal contacts [32].

In most of the previous studies evaluating online mental health services, further help-seeking was not considered the primary outcome (eg, [24,34,35]). Thus, it seems highly relevant to evaluate the impact of online services on further help-seeking behavior in adolescents with mental health concerns [36].

This study aimed to investigate the impact of counseling received from *krisenchat* on the further help-seeking behavior of young people, and to identify associated factors of further help-seeking, using anonymous data of chat users, including an anonymous online follow-up survey. Specifically, this study aimed to (1) determine the percentage of users of *krisenchat* who followed the recommendation for a referral to the health care system or to seek further help from a trusted adult person within 4 weeks; (2) investigate the facilitators and barriers to further help-seeking in *krisenchat* users who did and did not implement the recommendation, respectively; and (3) identify the factors associated with further help-seeking.

## Methods

### Participants and Procedure

*krisenchat* [37] is a German-speaking messenger-based psychosocial crisis chat counseling service for children, adolescents, and young adults under the age of 25 years. Counselors are volunteers with an education in psychology, psychotherapy, education, or social work and have received a minimum of 2 months of training in chat-based counseling and in screening for a potential clinical indication [32]. The aim of *krisenchat* is to listen to, calm, and comfort users during acute crises. Additionally, if deemed necessary, users also receive a recommendation to seek further help, either within the health care system (eg, professional local support services) or from a trusted adult person.

Usually, a chat counseling session consists of the following 5 phases: (1) building a trusting relationship, (2) understanding the problem, (3) clarifying the goal for the counseling session, (4) finding a common solution, and (5) properly ending the counseling session. For example, a female user aged 20 years and currently living with her violent parents may start out by mentioning an argument with her father (phase 1), but open up about being physically harmed after some empathic and supportive messages (phase 2). The counselor and user then agree that they want to do something about the situation involving outside help (phase 3). Since the user is not a minor anymore and not previously known to the youth office, this is not a case of child welfare endangerment. After asking the user what help she would like to receive and where she currently lives, the counselor informs her about her rights and options, and researches a local point of contact (in this case, an association called “Frauen Gegen Gewalt e.V.” [“Women Against Violence Association”]) (phase 4). Finally, the counselor and user agree on how to get in touch with the association and consensually end the chat after making sure that no open questions remain (phase 5).

Within the *krisenchat* counseling process, the first step toward a recommendation to a health service provider or a trusted adult person includes an assessment of age, gender, living conditions, crisis topic, (symptom) duration and severity, social resources, and existing affiliations to and previous experiences with help service providers. Usually, *krisenchat* users in need of further help are recommended to involve a trusted adult person from their private circle of acquaintances as a first low-threshold option for minor problems and conflicts. In cases where it seems necessary to eventually involve authorities (eg, youth welfare service), users are first recommended to involve a professional trusted adult person like a school psychologist. Other specialized professional counseling services with expertise in specific topics (eg, eating disorders, LGBTQ+, and drug abuse) are recommended when professional help seems necessary. Further, some users are recommended to seek medical help (eg, general practitioner and emergency ward) or psychotherapy treatment. When there are clear indicators of child welfare endangerment, contacting the youth welfare service is recommended.

For the purpose of this study, anonymous data from all chat users between October 1, 2021, and March 28, 2022, were extracted from the operational database. Data contained information collected by the counselors. In addition to this information, 2 online surveys were conducted: a feedback survey shortly after a chat session and a follow-up survey after 4 weeks.

For the feedback survey, each user received an automatically generated invitation via WhatsApp or SMS text messaging, which included a link to an online survey 6 hours after the first counseling session, if the chat session had at least 20 messages and the user was not considered at risk for child welfare endangerment by the psychological team. A minimum of 20 messages was established as an inclusion criterion based on the experiences of *krisenchat* with (1) the counseling process: after exchanging 20 messages counselors have usually gained enough information like age, gender, and the (primary) chat topic, which are necessary for a meaningful scientific evaluation of the chat; and (2) the user experience: after exchanging 20 messages the user has usually gained enough information to justify a feedback survey regarding their experience with the service.

For the follow-up survey, users received an automatically generated invitation via WhatsApp or SMS text messaging after 4 weeks, which included a link to an online survey, if the following criteria applied: the user had completed the feedback survey and had given their consent to be contacted again for the follow-up survey; a recommendation for a referral to the health care system or to seek further help from a trusted adult person had been given by the counselor and the counseling process was completed; and the user was not considered at risk for child welfare endangerment. Being considered at risk for child welfare endangerment was again an exclusion criterion in this step of the data collection process as these cases are often not labeled as cases of child welfare endangerment during the first counseling session, but only later in the counseling process. Children and young adults in these kinds of situations often contact *krisenchat* first with a minor problem to test if they can trust the service before opening up in a later counseling session about their real concern. If users attended more than one session, they received the link to the follow-up survey after the first recommendation for further help-seeking was made.

According to the definition used in a previous evaluation of *krisenchat* [32], a chat session was defined as a series of messages with no gaps between messages of more than 12 hours. It is possible that *krisenchat* users chat with more than one counselor during the course of one session. Users may or may not be made aware of the change in counselors, depending on the specific case.

Both surveys were set up in German using *typeform* [38]. At the beginning of each survey, it was offered to conduct the survey in simplified language in case users had limited German literacy.

**Figure 1:**
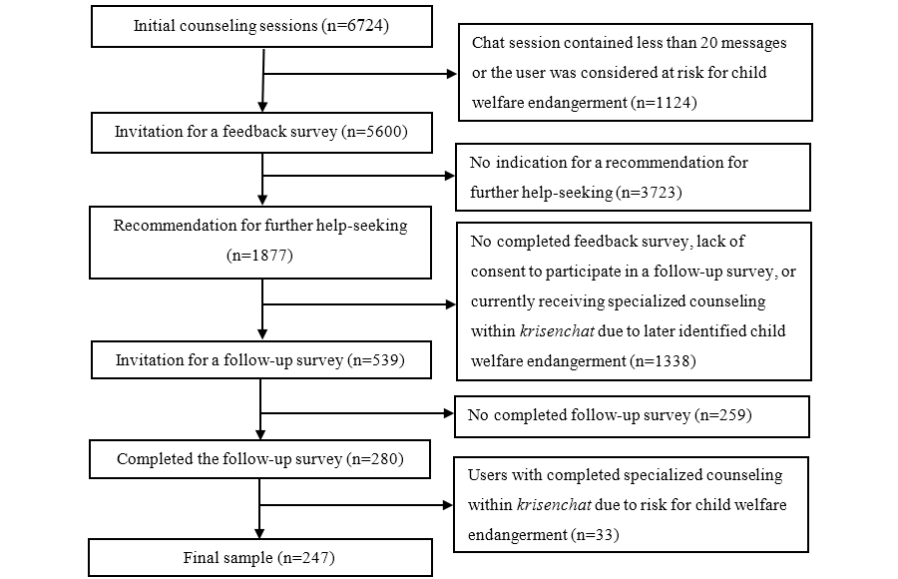
Flowchart of the data collection process.

Between October 1, 2021, and March 28, 2022, *krisenchat* received 10,614 requests in total. Of these, 3890 (36.6%) were excluded for one of the following reasons: users did not consent to the terms and conditions of *krisenchat*, they left the chat, or they did not receive a full counseling session due to a lack of capacity because of high demand. This left 6724 (63.4%) initial counseling sessions, of which 1124 (10.6%) were excluded as the chat session contained less than 20 messages (eg, when a user’s issue could be resolved within less than 20 messages) or the user was considered at risk for child welfare endangerment. The remaining 5600 (52.8%) users received an invitation for the feedback survey (see Figure 1 for the data collection process).

Of the 5600 users who received an invitation for the feedback survey, 539 (9.6%) later received an invitation for the follow-up survey. The other 5061 (90.4%) users were excluded from the follow-up survey as they met the exclusion criteria for the follow-up survey. In total, 280 (5.0%) users completed the follow-up survey, and of these, 33 (0.6%) had to be excluded due to technical circumstances, as they had completed specialized counseling within *krisenchat* due to being considered at risk for child welfare endangerment and had received the follow-up survey, although they met an exclusion criterion (ie, being considered at risk for child welfare endangerment). This resulted in a final sample of 247 users with completed feedback and follow-up surveys.

### Ethics Approval

Informed consent was provided online via an opt-in function before participating in each survey. Ethics approval was granted by the Ethics Committee of the Medical Faculty, University of Leipzig, on August 3, 2021 (file reference: 372/21-ek).

### Measures

#### Sociodemographic Information and Chat Use

Information regarding age and gender was collected by counselors if users disclosed them during a session. Further, counselors collected information on the topics of the counseling session. It was possible for a user to report several concerns at the same time, and counselors identified more than one chat topic for these users. For this analysis, the reported topics were aggregated into 4 categories: psychiatric symptoms, psychosocial distress, emotional distress, and violence (Table 1).

**Table 1:** Chat categories and topics.

Category	Included topics
Psychiatric symptoms	Anxiety, panic attacks, depressive symptoms or depression, obsessive-compulsive behavior, trauma (eg, flashbacks), depersonalization or derealization, substance abuse, behavioral addiction, symptoms related to borderline personality disorder, suicidality, nonsuicidal self-injury, psychotic symptoms, eating disorder symptoms, sleeping disorders, and psychosomatic complaints
Psychosocial distress	Conflicts in the family, at school, at work, in a relationship, or with friends; pressure to perform or high performing expectations (at school or at work, from oneself, or from others); difficulties adjusting to a new situation or environment; fear regarding the future; bullying; stress and overburden; online relationships; dealing with a relative’s or friend’s accident or physical illness; and dealing with a relative’s or friend’s suicide, nonsuicidal self-injury, mental disorder, or substance abuse
Emotional distress	Loneliness or isolation, lovesickness, feelings of guilt, low self-esteem, grief, difficulties in becoming independent from parents, emotional neglect, and anger or aggression
Violence	Being an offender or a victim of a criminal act, violence within the family or a relationship, and being a victim of physical or psychological violence

#### Feedback Survey

In the feedback survey, user well-being directly after the chat was assessed using 1 item (“How did you feel after the chat?”), answered on a 4-point Likert scale (1=“better,” 2=“rather better,” 3=“rather worse,” and 4=“worse”). A second item assessed the perceived helpfulness of the chat (“Was *krisenchat* able to help you with your concerns?”) on a 4-point Likert scale (1=“definitely,” 2=“rather yes,” 3=“rather no,” and 4=“not at all”).

#### Follow-up Survey

##### Further Help-Seeking, Facilitators, and Barriers

Users were asked to indicate which recommendation for a referral to the health care system or to seek further help from a trusted adult person they had received, including the following options: specialized professional counseling service, school psychologist or school social worker, social services (family assistance, educational assistance, or youth welfare service), local youth center, psychotherapist or social psychiatric service, general practitioner, calling an ambulance (to the hospital), parents, or another trusted adult person. If a user had received more than one recommendation, they were asked to indicate the option most relevant to them.

The actual further help-seeking behavior was assessed with the dichotomous item “After the counseling with *krisenchat*, did you contact the person or professional help service you were referred to?” Users who confirmed this item, indicating having followed the recommendation they received, were considered as displaying further help-seeking behavior. They were further asked if an appointment with the recommended service (or a talk with the recommended person) had already taken place, and if this was the case, it was assessed whether further appointments (or talks) were scheduled. The latter items were designed to measure the specific further help-seeking behavior based on the individual recommendation a user had received during the counseling process.

If users gave a positive answer (“yes”) on the item assessing actual further help-seeking behavior, 10 items on potential facilitators for help-seeking behavior were assessed in the following part. Likewise, in the case of a negative answer (“no”) on the item assessing actual further help-seeking behavior, 15 items on potential barriers for help-seeking behavior were assessed. Multiple choices were possible on each of the latter item sets. The respective items on facilitators and barriers to help-seeking were constructed based on a recent systematic review on help-seeking facilitators and barriers [19], and the practical experience of the counselors. Facilitators were assessed using 10 items grouped into 5 categories as follows: mental health literacy (3 items), symptom recognition (3 items), social support (1 item), improvement of self-efficacy through counseling (2 items), and an “other” category (1 item to indicate if reasons not covered by the other items applied). Barriers were assessed using 15 items grouped into the following 5 categories: stigmatization (2 items), negative family beliefs regarding help services (2 items), lack of mental health literacy (3 items), self-reliance and autonomy (3 items), structural factors (2 items), and an “other” category (3 items).

##### Self-efficacy

The General Self-efficacy Short Scale (German: “Allgemeine Selbstwirksamkeit Kurzskala [ASKU]” [39]) was used to assess the general efficacy beliefs of users. Self-efficacy relies on the definition by Albert Bandura, which describes people’s beliefs about their capability to execute behaviors that influence events affecting their lives [40]. The scale consists of 3 items representing single statements (eg, “I am able to solve most problems on my own”) with a 5-point Likert scale (1=“does not apply at all” to 5=“fully applies”), with higher mean scores indicating higher levels of self-efficacy. In this study, the scale showed an acceptable reliability with Cronbach α=.74. In other studies, the scale had a high reliability of ω=0.81 to 0.86 [39]. The mean reference values were 3.8 for low, 4.0 for medium, and 4.3 for high levels of self-efficacy [39].

### Statistical Analysis

Descriptive statistics were used for sociodemographic variables, recommendations (for a referral to the health care system or to seek further help from a trusted adult person), further help-seeking behavior, and facilitators and barriers to help-seeking. A chi-square test was used to analyze further help-seeking behavior depending on the recommended service or person. To this end, the number of categories of recommended services or persons was reduced by grouping them into the following 6 categories: psychotherapist or social psychiatric service, school psychologist or school social worker, parents, trusted adult person, counseling service, and other.

To analyze the associated factors of further help-seeking, the 2 subgroups of users displaying further help-seeking behavior and those not displaying further help-seeking behavior were compared regarding categorical variables (gender and session topic) using chi-square tests. For continuous variables (age, well-being after the chat, perceived helpfulness of the chat, and self-efficacy), both subgroups were compared using Mann-Whitney *U* tests owing to nonnormally distributed data, as indicated by Shapiro-Wilks tests (*P*<.05). Additionally, a logistic regression analysis was performed to analyze the association between various predictor variables (gender, age, session topic, well-being after the chat, perceived helpfulness of the chat, and self-efficacy) and further help-seeking behavior.

Bonferroni correction was used to account for multiple testing. To estimate effect sizes for chi-square tests, the ϕ coefficient was used, while Cramér *V* (ϕ_c_) was used when the contingency table was larger than 2×2, with ϕ_,_ ϕ_c_=0.10 indicating a small effect, ϕ_,_ ϕ_c_=0.30 indicating a medium effect, and ϕ_,_ ϕ_c_=0.50 indicating a large effect [41]. Effect sizes for Mann-Whitney *U* tests were interpreted as small (*r*<0.30), medium (*r*<0.50), and large (*r*>0.50) [41]. Statistical analyses were performed using IBM SPSS Statistics version 27 (IBM Corp). A 2-tailed α value of .05 was applied to statistical testing.

## Results

### Sociodemographic Characteristics

In the final sample of 247 users, most users were female (female users: 199/242, 82.2%; male users: 11/242, 4.5%; diverse users: 32/242, 13.2%; the data for 5 users were missing), and the mean user age was approximately 17 years (mean 17.31, SD 3.29; range 12-25 years). Nearly half of all users in each survey (feedback survey: 124/247, 50.2%; follow-up survey: 108/247, 43.7%) chose to conduct the survey in simplified language. The main reasons for contacting *krisenchat* were psychiatric symptoms (184/247, 74.5%), and psychosocial (136/247, 55.1%) or emotional distress (71/247, 28.7%). The mean self-efficacy score of *krisenchat* users was 2.82 (SD 0.86).

### Help-Seeking Behavior

The most frequently recommended services or persons to seek further help from included a psychotherapist or social psychiatric service (75/225, 33.3%), a school psychologist or school social worker (52/225, 23.1%), and the user’s parents (45/225, 20.0%). Nearly half of all users (120/247, 48.6%) indicated that they contacted the recommended service or person. A chi-square test indicated no significant differences in help-seeking behavior depending on the recommended service or person (χ^2^_5,225_=7.87; *P*=.16; ϕ_c_=0.19).

Of the 120 users who indicated having contacted the recommended service or person, 102 (85.0%) gave further information. Specifically, a large proportion stated that they already had an appointment (or talk) with the respective service or person (70/102, 68.6%) or that an appointment or talk was scheduled (17/102, 16.7%), while only 12 (11.8%) reported that there were no appointments available, 2 (2.0%) reported that they had contacted but not yet reached the recommended service or person, and 1 (1.0%) indicated being referred to another service. Of the 87 users who already had an appointment (or talk with a trusted person) or had scheduled an appointment (or talk), 75 (86.2%) provided further information on possible follow-up appointments. Specifically, the majority stated that a follow-up appointment (or talk) was already scheduled (48/75, 64.0%) or was planned but not yet scheduled (12/75, 16.0%), while 5 (6.7%) reported that they had already received sufficient help and another appointment or talk was not necessary and 1 (1.3%) indicated being referred to another service after the first appointment. The remaining 9 (12.0%) users reported that no follow-up appointments were available or that they did not want to schedule another appointment as they did not feel comfortable or had not received the help they needed.

### Facilitators and Barriers to Help-Seeking

A detailed description of the reported facilitators and barriers to further help-seeking is provided in Table 2. Users displaying further help-seeking behavior reported facilitators to help-seeking in the categories of mental health literacy (54/120, 45.0%), improvement of self-efficacy (55/120, 45.8%), symptom recognition (40/120, 33.3%), and social support (20/120, 16.7%). Users not displaying further help-seeking behavior reported barriers to help-seeking in the categories of stigmatization (60/127, 47.2%), lack of mental health literacy (59/127, 46.5%), self-reliance and autonomy (53/127, 41.7%), negative family beliefs regarding help services (53/127, 41.7%), structural factors (13/127, 10.2%), and other factors (eg, someone else has offered help; 24/127, 18.9%).

**Table 2:** Facilitators and barriers to help-seeking behavior (N=247).

Variable			Users displaying further help-seeking behavior (n=120), n (%)^a^	Users not displaying further help-seeking behavior (n=127), n (%)^a^
**Facilitators**				
	**Mental health literacy^b^**		54 (45.0)	N/A^c^
		Got new information	31 (25.8)	N/A
		Knowledge on where to seek further help	24 (20.0)	N/A
		Got a specific plan on how to seek further help	15 (12.5)	N/A
	**Self-efficacy^b^**		55 (45.8)	N/A
		Feeling encouraged to seek further help	53 (44.2)	N/A
		Feeling able to change the situation	10 (8.3)	N/A
	**Symptom recognition^b^**		40 (33.3)	N/A
		Seeing the problem more clearly	16 (13.3)	N/A
		Understood that what happened was not right	15 (12.5)	N/A
		Knowing how to talk about my problem/concern	17 (14.2)	N/A
	**Social support^b^**		20 (16.7)	N/A
		Feeling that someone is supporting me	20 (16.7)	N/A
	Other		0 (0.0%)	N/A
**Barriers**				
	**Stigmatization^b^**		N/A	60 (47.2)
		Fearing the reactions of others	N/A	52 (40.9)
		Not daring to talk with a stranger about serious concerns	N/A	23 (18.1)
	**Lack of mental health literacy^b^**		N/A	59 (46.5)
		Concerns will not be taken seriously	N/A	24 (18.9)
		Not deserving help, because problems are one’s own fault	N/A	31 (24.4)
		Fear of being admitted to a psychiatric ward	N/A	32 (25.2)
	**Self-reliance and autonomy^b^**		N/A	53 (41.7)
		Sought help elsewhere	N/A	15 (11.8)
		Can handle it alone	N/A	27 (21.3)
		Recommendation does not fit	N/A	14 (11.0)
	**Family beliefs^b^**		N/A	53 (41.7)
		Fear that parents will find out	N/A	42 (33.1)
		Professional help services are not trustworthy	N/A	20 (15.7)
	**Structural factors^b^**		N/A	13 (10.2)
		Referred help service is too far away or difficult to reach	N/A	2 (1.6)
		Have not had time yet	N/A	12 (19.4)
	**Other^b^**		N/A	24 (18.9)
		Someone else has offered help	N/A	6 (4.7)
		No help needed after all, already feeling better	N/A	8 (6.3)
		Other reasons, not specified	N/A	10 (7.9)

^a^Percentage is calculated from valid cases.

^b^Multiple answers were possible.

^c^N/A: not applicable.

### Associated Factors of Help-Seeking

Subgroup comparisons indicated significantly higher levels of self-efficacy (ASKU) in users displaying further help-seeking behavior than in those not displaying further help-seeking behavior (*P*=.01, small effect; Table 3). Moreover, users with further help-seeking behavior reported a higher perceived helpfulness of the chat than users without further help-seeking behavior (*P*=.02, small effect); however, this effect was not significant after Bonferroni correction. No significant group differences were found for gender, age, chat topics, and well-being after the chat (all *P*>.05). An overall logistic regression model for further help-seeking behavior was not statistically significant (χ^2^_10_=15.04; *P*=.13; Nagelkerkes *R*^2^=0.09). Only perceived helpfulness of the chat was significantly associated with further help-seeking behavior (B=0.56; *P*=.03; odds ratio 1.75, 95% CI 1.06-2.88). All other predictors in the model were not significant (all *P*>.05).

**Table 3:** Group comparison between users with and those without further help-seeking behavior (N=247).

Variable			Users displaying further help-seeking behavior (n=120)		Users not displaying further help-seeking behavior (n=127)		Chi-square (*df*)		*U*		*P* value		Effect size
**Gender, n (%)^a^**							2.94 (2,242)		N/A^b^		.23		ϕ_c_^c^=0.11
	Female	94 (79.0)		105 (85.4)									
	Male	8 (6.7)		3 (2.4)									
	Diverse	17 (14.3)		15 (12.2)									
Age (years), mean (SD)			17.68 (3.62)		16.97 (2.91)		N/A		6759.50		.22		*r*=0.08
Topic: Psychiatric symptoms, n (%)^a^			86 (71.7)		98 (77.2)		0.98 (1,247)		N/A		.32		ϕ=0.06
Topic: Psychosocial distress, n (%)^a^			62 (51.7)		74 (58.3)		1.09 (1,247)		N/A		.30		ϕ=0.07
Topic: Emotional distress, n (%)^a^			32 (26.7)		39 (30.7)		0.49 (1,247)		N/A		.48		ϕ=0.05
Topic: Violence, n (%)^a^			4 (3.3)		3 (2.4)		0.21 (1,247)		N/A		.65		ϕ=0.03
Well-being score, mean (SD)			2.39 (0.65)		2.25 (0.65)		N/A		6029.50		.10		*r*=0.11
Perceived helpfulness score, mean (SD)			2.41 (0.68)		2.22 (0.68)		N/A		6303.50		.02		*r*=0.15
Self-efficacy score (ASKU^d^), mean (SD)			2.97 (0.82)		2.69 (0.88)		N/A		5797.00		.01		*r*=0.17

^a^Percentage is calculated from valid cases.

^b^N/A: not applicable.

^c^ϕ_c_: Cramér *V*.

^d^ASKU: Allgemeine Selbstwirksamkeit Kurzskala (“General Self-efficacy Short Scale”).

## Discussion

### Summary

Overall, the results imply that children and young adults using *krisenchat* experience benefits. The 24/7 online counseling service may facilitate seeking further professional help within the health care system or support from others.

It is important to highlight that the referral of users to professional help services or personal contacts is only one of the aims of *krisenchat* (only in 27.9% of all *krisenchat* users), along with listening, calming, and comforting in an acute crisis; engaging in collaborative problem-solving; emphasizing and supporting chat users to find their own solutions; and improving mental health literacy [32]. Therefore, the current results only refer to a subgroup of all chat users (for more details on the overall use of *krisenchat* see [32]).

### Help-Seeking Behavior

The results of this study show that children and young adults using *krisenchat* benefit in terms of seeking further help. Nearly half of all users (120/247, 48.6%) indicated that they contacted the recommended service or person after the professional counseling. This percentage appears to be very high and shows *krisenchat* to be an important gatekeeper service for young people and adolescents. Nevertheless, owing to novelty, there are at present limited studies on the evaluation of online chat counseling or crisis text line interventions. The few available studies are not comparable to our study owing to different foci or differing measurement approaches (eg, evaluation of users’ perceptions of effectiveness right after the chat or focus on suicide preventive aspects only [42-44]).

Nevertheless, more than half of the users who took part in the follow-up survey reported that they did not seek further help. There were no differences in help-seeking behavior depending on the recommended service or person. Other potential reasons for not displaying further help-seeking behavior remain unclear, and further research is needed in this area.

As mentioned earlier, little is known about the effects or benefits of online counseling or helplines, since studies are methodologically challenging not least because of the anonymity of the services. In a recent systematic review on the state of youth helplines, which is at least partly comparable to the *krisenchat* service, the authors concluded that helplines may provide a beneficial service to youth and that psychosocial concerns are the main reasons for contacting these services [45]. This result is in line with previous evaluations of *krisenchat* [32,33]. The authors also concluded from the results of the review that there is a lack of literature owing to a lack of controlled trials on the one hand, and complex methodological/ethical barriers preventing such trials on the other [45]. Few studies have investigated long-term outcomes of crisis hotlines in the past, mainly focusing on suicide rates within a population [46,47].

### Facilitators and Barriers to Help-Seeking

Nearly half of the users who sought further help reported mental health literacy to be an important facilitator, and reported the improvement of self-efficacy and symptom recognition as an effect of using the chat counseling service. In users who reported no further help-seeking behavior, the most frequent barriers were stigmatization, lack of mental health literacy, the need for self-reliance and autonomy, and negative family beliefs regarding help services. In line with the literature, the results also indicate that improving mental health literacy seems to be a key strategy for improving help-seeking in adolescents and young adults [19,48-50]. There are different approaches for improving mental health literacy in young people, including whole-of-community campaigns; community campaigns aimed at a youth audience; school-based interventions teaching help-seeking skills, mental health literacy, or resilience; and programs training individuals to better intervene in a mental health crisis [51,52]. 

Overall, it is important to highlight again that previous studies revealed that *krisenchat* may lower the barriers to access help, that is, about 50% of those contacting *krisenchat* reported that they never had contact with the professional health care system before [32].

### Associated Factors of Help-Seeking

The analysis on group differences revealed that users displaying further help-seeking behavior reported higher levels of self-efficacy compared with those not displaying further help-seeking behavior, but both subgroups did not differ in gender, age, recommended service or person, chat topics, perceived helpfulness of the chat, and well-being after the chat. Generally, the mean self-efficacy score of all *krisenchat* users was 2.82 (SD 0.86), which is considered to be very low [39]. In the logistic regression analysis, only a higher perceived helpfulness of the chat was associated with further help-seeking behavior; however, the interpretability of this result is limited as the overall model was not significant.

Surprisingly, in this study, central aspects of the *krisenchat* user (age, gender, and well-being) and service usage (chat topic and recommended service or person) seemed to be irrelevant for predicting further help-seeking. This means, in turn, that based on recent data, the only factor relevant for further help-seeking is self-efficacy. In line with the recent literature, this result supports the claim for more research regarding help-seeking in youth and adolescents, in order to be able to target interventions to improve and facilitate help-seeking behavior [19].

### Strengths and Limitations

This study is the first to examine the impact of using *krisenchat* on the further help-seeking behavior of young people, and to identify associated factors of further help-seeking. However, a number of limitations must be considered. The final sample used for this analysis consisted of only 13.2% of all users of the service, who received a recommendation for a referral to the health care system or to seek further help from a trusted adult person, with completed feedback and follow-up surveys. There might be further associated factors of help-seeking or different patterns in the help-seeking behavior of users, which could not be detected due to this attrition rate. Additionally, there might be key differences between those who filled in the survey and those who did not (eg, regarding motivation, perceived helpfulness of the chat, chat topics, and other aspects). Further, several other variables, such as cultural background, fluency in the German language, education level, and socioeconomic status, which could not be assessed due to the anonymity of *krisenchat*, might influence help-seeking behavior and factors associated with further help-seeking. A further limitation includes the use of self-report data for assessing the recommendation for further help-seeking.

Nevertheless, given the fact that there are limited studies on the impact or benefit of 24/7 online counseling, the sample size of 247 is reasonable and can serve as a basis for stimulating further research and advances in measurements.

### Conclusion

The results of this study suggest that children and young adults using *krisenchat* may benefit in terms of seeking further help. Further help-seeking seems to be associated with higher levels of self-efficacy. Further research is necessary to better understand how young people can further benefit from 24/7 online counseling. Longitudinal research seems especially crucial to understand who benefits when and from what kind of service and to assess which factors are associated with help-seeking behavior.

## Abbreviations

ASKU: Allgemeine Selbstwirksamkeit Kurzskala (“General Self-efficacy Short Scale”)
